# Mild cognitive impairment and neuropsychological examination

**DOI:** 10.3389/fpsyg.2025.1662151

**Published:** 2025-11-06

**Authors:** Erik Hessen

**Affiliations:** Department of Neurology, Akershus University Hospital and Department of Psychology, University of Oslo, Oslo, Norway

**Keywords:** MCI, neuropsychology, dementia, memory, cognition

## Abstract

**Objective:**

Mild cognitive impairment (MCI) is a condition that involves impairment of cognitive function beyond what is expected with normal ageing. The condition is prevalent in old age and may be a risk factor for the development of dementia. However, MCI can have medical and psychological causes that do not cause further cognitive decline or dementia. Thus, it is important to identify MCI at an early stage, aiming to prevent further impairment, to inform necessary life adaptation to cognitive problems or to treat the condition when the cause of cognitive impairment can be treated.

**Method:**

The present paper is not based on a comprehensive review of the field but considers the various types of MCI according to the internationally prevailing diagnostic systems and algorithms, proposed key progression factors, focusing on the role of neuropsychological assessment in the diagnosis of MCI.

**Results:**

The paper discusses according to prevailing diagnostic systems and algorithms, which cognitive domains that are relevant to investigate, which tests that may be relevant, what kind of norms have satisfactory quality, which cut-off scores do best balance sensitivity and specificity in a neurodiagnostic context, and what kind of conclusions and recommendations that can be drawn from neuropsychological findings.

**Conclusion:**

Comprehensive neuropsychological assessment based on more than one test in each of the five cognitive domains (memory, attention, language, visuospatial function, and executive function) recommended by NIA-AA, DSM-5 and ICD-11, employing national and culturally adapted norms has shown superior validity regarding neuropathology and prognosis and is recommended as best practice.

## Mild cognitive impairment

Mild cognitive impairment (MCI) is a condition that involves cognitive impairment beyond that expected with normal aging. Cognitive impairment can manifest itself in various ways. Most often reported are difficulties with memory, difficulty in coming up with words and expressions, problems with spatial orientation, a sense of reduced spatial awareness and sometimes difficulties with thinking, problem solving and judgment (Winblad et al., 2004). Many with MCI have insight into their problems, while others have limited understanding of cognitive impairment (e.g., Edmonds et al., 2014; Ilardi et al., 2025). The cognitive and behavioral changes seen in MCI are not so pronounced that the ability to cope with work or the challenge of daily life is significantly reduced (Albert et al., 2011). To maintain good health and quality of life in old age, it is important to identify definite cognitive impairment at an early stage with a view to preventing further impairment, adapting to life in relation to the identified impairment, or to initiate treatment where the cause of cognitive impairment can be treated.

## Occurrence

A recent comprehensive review found that the global prevalence of mild cognitive impairment in the geriatric population (above 65 years) is 23.7% (Salari et al., 2025). In a representative Norwegian study, the prevalence of dementia and mild cognitive impairment in people over 70 years of age was estimated at 16.2 and 35.6%, respectively, suggesting that around every second person over 70 years of age in Norway has mild cognitive impairment or dementia (Gjøra et al., 2023).

## Subtypes and prognosis

MCI can be caused by various diseases that affect the brain. Alzheimer’s disease is the most common cause of MCI and dementia. The second most common cause of MCI and dementia is cerebrovascular disease/vascular dementia, followed by Parkinson’s disease and other neurodegenerative conditions such as dementia with Lewy bodies and frontotemporal dementia. MCI and cognitive problems can also be associated with other conditions, including affective disorders, stress, chronic pain, fatigue, sleep disorders, low vitamin levels (especially B-12), traumatic brain injury, various neurological diseases, side effects of various medications, and alcohol or other drug abuse (American Psychiatric Association, 2013).

Because MCI and cognitive problems can be influenced by many conditions and causes, the prognosis also varies. MCI can increase the risk of dementia, but not everyone with MCI develops increased cognitive impairment. Some people remain in a state of stable mild cognitive impairment and for some, cognitive function improves to such an extent that they no longer have cognitive impairment. Several of the causes of cognitive impairment and cognitive difficulties are reversible and can be treated, such as depression or sleep problems. Cholinesterase inhibitors are medications that are used to try to slow down cognitive impairment in people with Alzheimer’s dementia. Studies suggest that such medications may also have some effect on slowing the development of MCI when it is caused by Alzheimer’s disease, but due to the possibility of significant side effects, such treatment is not currently recommended as routine treatment for MCI caused by Alzheimer’s disease (Matsunaga et al., 2019). In February 2025 EMA’s human medicines committee (CHMP) recommended a new drug Leqembi (Leqanenmab), a monoclonal antibody as treatment for mild cognitive impairment (MCI) due to Alzheimer’s disease (AD). Leqembi targets amyloid plaques aiming to reduce their buildup in the brain. The drug had already been approved for use by the U.S. Food and Drug Administration (FDA) on July 6, 2023. Some clinical trials have indicated that Leqembi may slow the progression of cognitive decline in people with early stages of Alzheimer’s or MCI, particularly in memory and executive function (e.g., Iwatsubo, 2023). While the drug represents an exciting step in Alzheimer’s treatment, especially for patients with MCI in the early stages its long-term effectiveness, possible side effects as well as cost are still areas of concern. In the case of cerebrovascular causes of MCI, there are intervention options. It has been shown that treating high blood pressure as part of vascular disease and a risk factor for vascular dementia can significantly reduce the risk of mild cognitive impairment (Williamson et al., 2019). The annual conversion rate from MCI to dementia for patients with a mean age of 74 years is around 10%, although with significant variation between different studies, while the annual conversion rate to dementia for the same age group in the normal population is estimated to be between 1 and 2% (Bruscoli and Lovestone, 2004). Many studies show that people who have been diagnosed with MCI can also develop improved or normalized cognitive function during follow-up. For example, Overton et al. (2019) found in a large Swedish study of people between 60 and 95 years of age that as many as 58% of those who were considered to have MCI at inclusion had completely normalized cognitive function at 6 years of follow-up. In another Swedish-Norwegian study of patients from memory clinics with an average age of 63 years, normal cognitive function was found at follow-up after 2 years in 25% of those who had MCI at inclusion in the study (Hessen et al., 2014). Because many different medical and psychological conditions can contribute to MCI, the appropriate treatment for MCI will vary, depending on the underlying etiology. Thus, a satisfactory discussion on the treatment of MCI must take into consideration all the possible etiologies, requiring a very comprehensive discussion, beyond the scope of this paper focusing on neuropsychological assessment of the condition.

The original criteria for MCI (called the Mayo Clinic Core Criteria) were published in 1999 (Petersen et al., 1999). The criteria emphasized memory impairment rather than other cognitive domains. After a few years, these criteria were revised because it was well known that Alzheimer’s disease and other forms of neurodegenerative diseases also affect cognitive domains other than memory. The revised Mayo Clinic criteria (also called the Winblad criteria) (Winblad et al., 2004) have proven to be clinically useful over time. The criteria have subcategories based on which cognitive domains are affected. In addition to providing a better functional description, the rationale for subcategorizing MCI was an attempt to produce clinically meaningful categories associated with different etiologies. According to the Winblad criteria, MCI is roughly divided into 4 categories:

- (1) Amnestic single-domain or (2) amnestic multiple-domain MCI.
- (3) Non- amnestic single-domain or (4) non- amnestic multiple-domain MCI.

**Figure 1:**
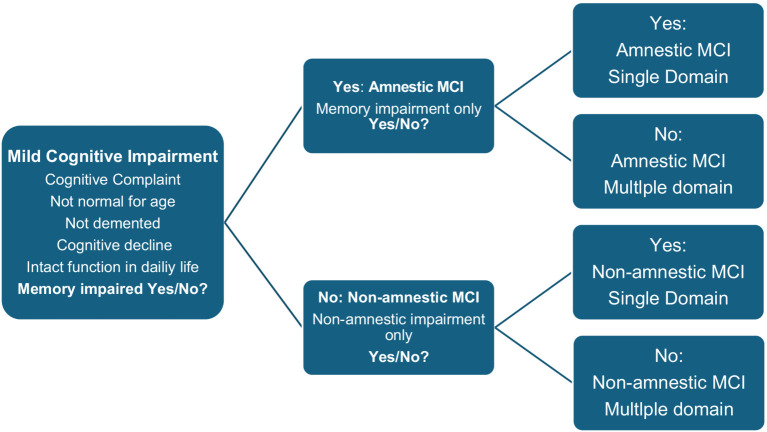
The diagnostic process and the cognitive subcategories according to the Winblad criteria (Winblad et al., 2004).

The diagnostic process and the cognitive subcategories according to the Winblad criteria (Winblad et al., 2004) are illustrated in Figure 1:

Subsequent studies have compared the progression of cognitive impairment based on the different subtypes of the Winblad criteria and found that amnestic multiple-domain MCI better predicted progression to Alzheimer’s dementia than amnestic single-domain or non- amnestic MCI, with annual progression rates ranging from 4 to 25% (Bradfield and Ames, 2020; Petersen et al., 2009; Weiner et al., 2013). Current knowledge suggests that amnestic MCI outperforms non- amnestic MCI, and that amnestic multiple-domain MCI outperforms amnestic single-domain MCI in predicting progression to Alzheimer’s dementia. The reason for this is uncertain, but one possible explanation is that the presence of impairment also in cognitive domains other than just memory may reflect a more advanced stage of the disease that is closer to the development of dementia.

## Neuropsychology and diagnostics of mild cognitive impairment

One of the most important tasks of neuropsychology is to detect and classify abnormal cognitive function associated with organic brain-related impairment (Lezak et al., 2004). Consequently, neuropsychology as a field is centrally located in mapping early stages of neurodegenerative conditions that affect cognition. In this context, crucial questions are which cognitive domains are relevant to investigate, which tests are most relevant, what kind of norms have satisfactory quality and which cutoff values best balance sensitivity and specificity about organic brain-related cognitive impairment. These topics are discussed in this article.

## Diagnostic criteria for MCI

There are several central systems for diagnosing MCI developed by different professional communities and organizations (Albert et al., 2011; American Psychiatric Association, 2013; World Health Organization, (2019/2021); Jak et al., 2009). There are significant similarities between the systems, but also differences with different focus on cognitive subtypes, different thresholds for cognitive impairment and different breadth of the examination. The review first summarizes the common features. Then, the cognitive/neuropsychological examination recommended in each of the systems is reviewed individually.

Common elements in diagnostic criteria to the National Institute on Aging and the Alzheimer’s Association (NIA-AA) (Albert et al., 2011), American Psychiatric Association (2013), and World Health Organization (2019/2021).

- There must be a concern about a change in cognitive function compared to previous function, communicated by the patient, a close informant or an experienced clinician.
- The person functions independently, experiences minimal difficulty performing tasks of daily living such as paying bills, cooking, and shopping, does not have significant impairment in social or occupational functioning, and does not have dementia.
- The cognitive impairment cannot be explained by delirium or a transient state of confusion.
- Differential diagnosis: The cognitive impairment should not be better explained by another mental or physical disorder. In the DSM-5 and ICD-11 criteria, it should be specified whether the condition is caused by Alzheimer’s disease, frontotemporal degeneration, Lewy body disease, vascular disease, traumatic brain injury, substance or medication use, HIV infection, prion disease, Parkinson’s disease, Huntington’s disease, another medical condition, multiple etiologies, or has an unspecified cause.

## Cognitive examination according to NIA-AA criteria

According to the NIA-AA (Albert et al., 2011), which is the prevailing and dominant criterion for diagnosing MCI, impairment is required in one or more of the following cognitive domains: attention, memory, language, visuospatial skills, and executive function. The NIA-AA criteria do not specify specific tests that should be used. Impairment is defined as test scores 1–1.5 standard deviations below expected based on age- and education-corrected norms. Typical of the assessment at many memory clinics is that the patient is assessed with short test batteries, often only one test within each of the cognitive domains.

## Cognitive examination according to DSM-5 criteria

American Psychiatric Association (2013) has changed terminology and uses the term mild neurocognitive disorder instead of MCI. Likewise, the term severe neurocognitive disorder is used instead of dementia.

DSM-5 uses the same five cognitive domains as the NIA-AA criteria but has added social cognition as a sixth domain (Sachdev et al., 2014). Examples of symptoms and typical observations are provided for each domain, but specific tests are not recommended. Social cognition was added because some neurocognitive conditions are associated with socially inappropriate behavior. Previous diagnostic criteria have typically used the term personality change.

According to DSM-5, there should be modest impairment in cognitive function, preferably documented by standardized neuropsychological testing, or in the absence of this, documented by another quantified clinical assessment.

Norms adapted to the patient’s age, education, and cultural background are part of the standard assessment of neurocognitive disorder and are of particular importance in the assessment of mild neurocognitive disorder (MCI). For severe neurocognitive disorder (dementia), test performance is typically 2 standard deviations or more below the mean (3rd percentile or lower). For mild neurocognitive disorder (MCI), test performance is typically between 1 and 2 standard deviations below the mean (16th and 3rd percentile).

## ICD-11 criteria

The ICD-11 criteria have been launched by the World Health Organization (2019/2021) and will in the future be the official international diagnostic system. The criteria for MCI, and current differential diagnoses, are very much based on DSM-5, and are not reproduced in detail here. ICD-11 also requires objective evidence of cognitive impairment based on standardized neuropsychological/cognitive testing or, in the absence of this, other quantified clinical assessment.

## Comprehensive neuropsychological diagnostic criteria for MCI

Conventional MCI diagnostics using diagnostic criteria are often based on short test batteries, often with only one test for each cognitive domain, where failure in a single test measure in addition to subjective cognitive impairment and clinical assessment can provide the basis for an MCI diagnosis. Diagnostic stability and predictive accuracy are known problems when MCI is diagnosed using this method (Mitchell and Shiri- Feshki, 2009; Edmonds et al., 2015; Edmonds et al., 2016; Edmonds et al., 2019; Bondi et al., 2014). This approach has been shown to be vulnerable to false positive diagnoses (Clark et al., 2013), has shown limited sensitivity to biomarkers for Alzheimer’s disease (Edmonds et al., 2015; Edmonds et al., 2016) and uncertain prediction of Alzheimer’s disease (Edmonds et al., 2016).

The classical neuropsychological method involves a broader examination using multiple tests in the same cognitive domain and interpretation based on patterns in the test results (Lezak et al., 2004). With this as a starting point, Jak et al. (2009) developed an MCI criterion based on a more comprehensive neuropsychological examination. The criterion was based on Taylor and Heaton (2001) who had found that a neuropsychological test score of 1 SD below the mean represented the best cut-off value for distinguishing between neurologically healthy and neurologically ill people. Such a liberal failure criterion is problematic in practice because it is common for neurologically healthy adults to achieve one and often several test scores below this level when tested with a standard neuropsychological test battery (Heaton et al., 2004; Binder et al., 2009) However, it is much less common to have two test scores in the same cognitive domain below such a cut-off value. For example, Palmer et al. (1998) found that fewer than 5% of neurologically healthy older adults had two or more impaired scores in the same cognitive domain. Other studies point in the same direction. For example, Grewal et al. (2023) found high incidence of multiple low scores in cognitively intact patients at a memory clinic. They argue for increased attention to this and the establishment of baseline rates for low scores that can be used in the assessment to reduce the number of false positive diagnoses.

Based on this knowledge, Jak et al. (2009) tested neuropsychological criteria for identifying MCI. The failure criterion was that at least two tests within one cognitive domain should be below the cutoff value for that domain to contribute to the MCI classification. To best balance specificity and sensitivity, they chose a cutoff value of 1 SD below the mean based on demographic norms (Heaton et al., 2004). Accordingly, a person was classified as normally functioning if only one test performance in a cognitive domain was below 1 SD below the mean.

Validation of the Jak/Bondi criteria (Jak et al., 2009) demonstrated neuropsychological heterogeneity beyond the amnestic/non- amnestic MCI distinction proposed in the Winblad criteria (Winblad et al., 2004). MCI profiles with an emphasis on memory difficulties, naming difficulties, executive difficulties, mixed MCI profiles were found and in addition, use of these criteria has demonstrated cognitive normality in many who receive a false-positive MCI diagnosis based on brief conventional MCI examinations (Clark et al., 2013). Diagnostics according to the comprehensive criteria have also shown better association with biomarkers for Alzheimer’s disease and significantly more accurate prediction of cognitive function over time, including progression to Alzheimer’s disease, than conventional MCI criteria (Bondi et al., 2014; Edmonds et al., 2016; Edmonds et al., 2020; Eliassen et al., 2017).

## Financial capacity

Lack of ability to deal with finances and money can affect people’s quality of life and are associated with mild cognitive impairment and more severe forms of cognitive impairment. In clear cases of MCI, it is therefore important to assess the financial capacity of the patient. Despite the importance of evaluating this, only a few measures have been developed to assess this problem. A useful tool which is used in Europe is the Legal Capacity for Property Law Transactions Assessment Scale (LCPLTAS) which highlights that deficits are not only numerical but more complex in older MCI patients (Giannouli et al., 2018). Another tool, the Numerical Activities of Daily Living – Financial (NADL-F) test is described in Arcara et al., 2017. The test is designed to assess financial capacity in patients with cognitive problems. Both tests cover important activities involving financial capacities in daily life. They have shown satisfactory psychometric properties and good validity for measuring financial abilities. These or other methods for assessment of financial capabilities are recommended as part of standard assessment of patients with MCI or dementia.

## Biological progression factors

It is important that an MCI diagnosis is accompanied by an etiological investigation with a view to known biological progression factors for degenerative conditions. Different neurodegenerative conditions may be associated with different cognitive profiles, but none of these conditions have completely specific profiles. A typical amnestic syndrome may lead to suspicion of underlying Alzheimer’s disease. If the patient has problems with attention, concentration and visuospatial function, underlying dementia with Lewy bodies may be relevant to consider, while a patient with major behavioral changes, lack of insight, apathy and problems with attention and concentration may be in an early stage of frontotemporal dementia. Vascular-related cognitive impairment may be amnestic, non- amnestic or a combination of these.

**Figure 2:**
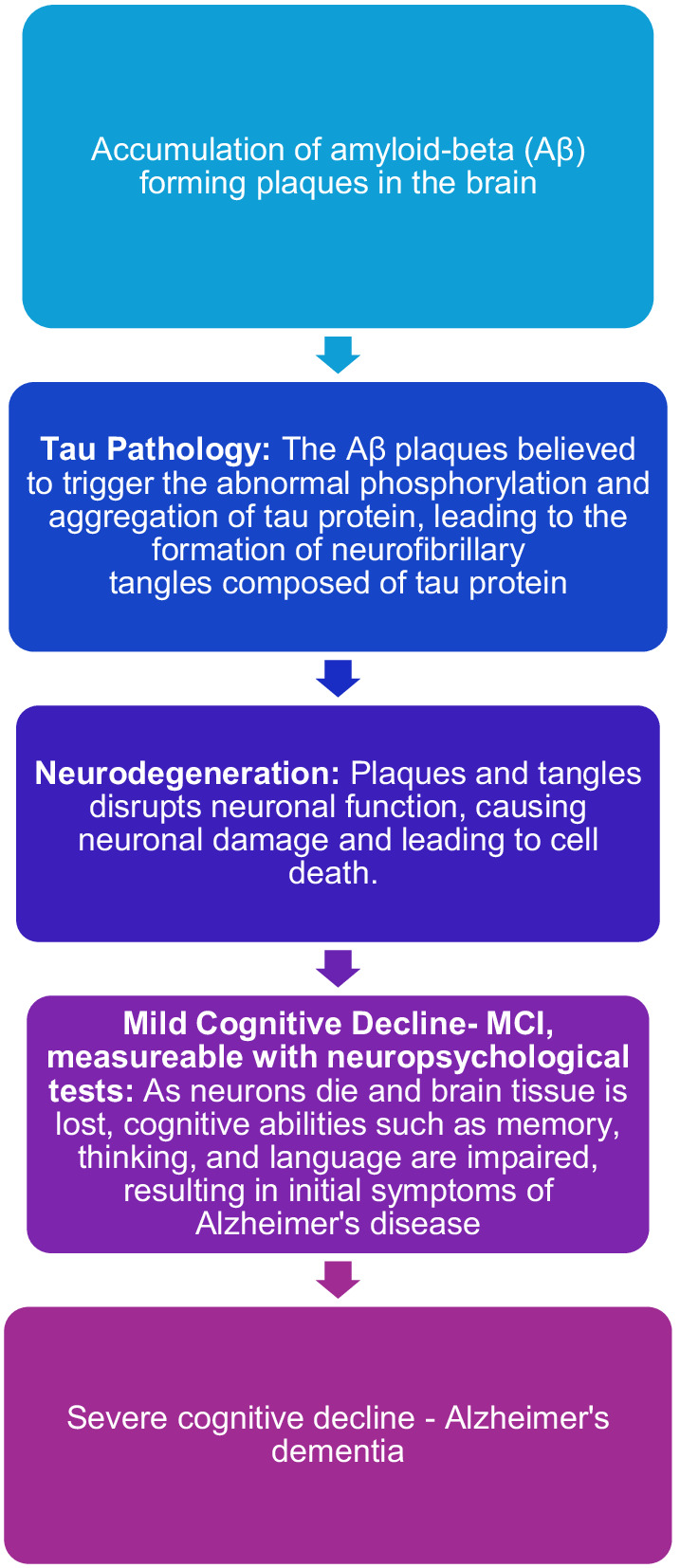
Visualization of the proposed model for the pathogenesis of Alzheimer’s disease (Jack et al., 2010).

Alzheimer’s disease is the cause of most cases of neurodegenerative cognitive impairment and dementia. There is evidence that biological changes associated with this disease begin many years, perhaps 10–15 years, before it is possible to register the first symptoms. There are different views on what kind of biological events are most likely to lead to the development of Alzheimer’s disease. The table below illustrates a central hypothesis about the disease stages in Alzheimer’s disease (Jack et al., 2010). This hypothesis assumes that the formation of amyloid deposits in the brain can have a neurotoxic effect that leads to the development of Alzheimer’s disease and later dementia. Relevant biomarkers for amyloid deposits in the brain (amyloidosis) are low values of the protein beta- amyloid 42 (Aβ 42) in spinal fluid and elevated levels of amyloid PET in the brain. According to the hypothesis, it is thought that, after a period that varies from person to person, neuronal dysfunction and neurodegeneration will gradually develop to become the dominant pathological process. The central biomarkers for neuronal damage are increased occurrence of tau proteins in spinal fluid and signs of cerebral atrophy that can be seen on cerebral MRI. Roughly parallel to the fact that biomarkers for neuronal damage become measurable, it is thought that cognitive symptoms will become noticeable, perhaps first in the form of subtle subjective changes and later in the form of MCI that can be objectified with neuropsychological findings according to current diagnostic criteria. However, the hypothesis has been criticized, among other things because many studies have not found a correlation between cognitive impairment and amyloid deposition. It has long been known that carriers of the genotype apolipoprotein E4 (APOE-4) have a higher risk of faster progression to dementia. Around 25% of the population has one copy of APOE-4, which can both contribute to lowering the age of onset and increase the risk of developing Alzheimer’s dementia. Only 2–3% of the population have two copies of APOE-4, which can significantly increase the risk of developing Alzheimer’s dementia (Sienski et al., 2021). In the evaluation of MCI, it is therefore important to map APOE-4 status, but in clinical practice, knowledge of APOE status usually contributes less to the diagnostic assessment than mapping cognitive function, brain imaging, and mapping markers of amyloid deposition and neuronal damage (Weiner et al., 2015). All these predictors are relevant for people who can be said to be on the Alzheimer’s spectrum. There are also known biomarkers for other degenerative conditions, but many of these markers are currently less certain than markers for Alzheimer’s disease (Figure 2).

## Model for neuropsychological examination in the assessment of MCI

All the diagnostic systems described require neuropsychological/cognitive testing within five core cognitive domains: memory, attention, language, visuospatial function, and executive function. DSM-5 and ICD-11 also include social cognition (personality change) as a sixth cognitive domain. This is not specifically discussed in this context as methods and tests for detecting impairments in social cognition/personality change represent a somewhat different approach than the five traditional cognitive domains mentioned above. Neither DSM-5 nor ICD-11 recommend specific tests to measure functioning in the five traditional cognitive domains, which all the diagnostic systems rely on.

The neuropsychological method (Jak et al., 2009) has been validated against biomarkers and progression of cognitive impairment. Regardless of which tests have been used, regardless of age groups and geographical area, similar results have been obtained with regard to sensitivity, specificity and longitudinal prediction, which is considered to provide further support for this diagnostic algorithm for MCI (Jak et al., 2009; Bondi et al., 2014; Eliassen et al., 2017; Wong et al., 2018; Edmonds et al., 2020). Table 1 provides an overview of cognitive domains (NIA-AA criteria, DSM-5 and ICD-11) and tests used in the validation of the Jak/Bondi criteria for MCI (Jak et al., 2009).

**Table 1:** Cognitive domains (NIA-AA criteria, DSM-5 and ICD-11) and tests used in testing the neuropsychological diagnostic criteria for MCI (Jak et al., 2009).

Learning/memory	Attention	Language	Visuospatial function	Executive function
Tests used in testing the neuropsychological diagnostic criteria for MCI (Jak et al., 2009; Edmonds et al., 2020; Wong et al., 2018; Eliassen et al., 2017)				
Logical Memory I and II- (WMS) Visual Recall I and II-(WMS) California Verbal Learning Test (CVLT) Rey Auditory Verbal Learning Test (RAVLT) (for both CVLT and RAVLT: learning 1–5, delayed free recall, and delayed recognition)	Number span forward (WAIS) Trail Making Test - A Coding – (WAIS)	Boston Naming Test Phonemic fluency: FAS (Controlled Oral Word Association Test- COWAT) Semantic flow: Animals (COWAT) Similarities (WAIS)	Block design (WAIS) Clock drawing Hooper Visual Organization Test Complex figure copying (Rey Complex Figure Test-RCFT)	Trail Making Test - B Number span backwards (WAIS) Wisconsin Card Sorting Test −48- short version Color-Word Interference Tests (inhibition and alternation) Delis-Kaplan Executive Function System-D-KEFS

References for tests: WMS: Wechsler D. Wechsler Memory Scale - Revised. New York: Psychological Corporation (1987). CVLT: Delis DC, Kramer JH, Kaplan E, and Ober B A. California Verbal Learning Test--Second Edition (CVLT –II). APA PsycTests (1987–2000). RAVLT: Schmidt M. Rey auditory verbal learning test: A handbook. Los Angeles, CA: Western Psychological Services (1996). WAIS: Wechsler D. Wechsler Adult Intelligence Scale--Fourth Edition (WAIS-IV). APA PsycTests (2008). Trail Making Test A and B: Reitan RM, Wolfson D. The Halstead Reitan Neuropsychological Test Battery. 2nd ed. Tucson (AZ): Neuropsychology Press (1993). Boston Naming Test: Kaplan EF, Goodglass H, Weintraub S. The Boston Naming Test. Philadelphia: Lea and Febiger (1983). Hooper visuals organization test: Hooper HE. Hooper Visual Organization Test. Los Angeles: Western Psychological Services (1983). COWAT: Benton AL, Hamsher de SK and Sivan AB. Controlled Oral Word Association Test (COWAT) (1983). RCFT: Meyers JE and Meyers KR. Rey Complex Figure Test and Recognition Trial: Professional Manual. Psychological Assessment Resources, Odessa (1995). Clock drawing: Freedman M, Leach L, Kaplan E, Winocur G, Shulman KI, and Delis DC. Clock drawing: A neuropsychological analysis. Oxford University Press (1994). Wisconsin Card Sorting Test: Heaton RK, Chelune GJ, Talley JL, et al. Wisconsin Card Sorting Test manual. Odessa, FL: Psychological Assessment Resources, Inc (1993). D-KEFS: Delis DC, Kaplan E, and Kramer JH. Delis-Kaplan Executive Function System (D–KEFS), APA PsycTests (2001).

## Use of cognitive tests as indirect measures of initial cognitive functioning level

When diagnosing MCI, according to the central diagnostic systems, there must be evidence of cognitive reduction from a previous level of functioning within at least one of the relevant cognitive domains. Various procedures are specified for the assessment of cognitive impairment, where information from the patient and relatives is central. Likewise, there must be documentation of impairment based on standardized neuropsychological testing. From the perspective of neuropsychology, it is striking that none of the prevailing diagnostic criteria, nor those developed by neuropsychologists (Jak et al., 2009), mention the use of tests that can provide an indication of the original/premorbid cognitive level of functioning as a necessary part of the neuropsychological examination when asking about impairment in cognitive function in relation to the previous level of functioning. The use of such tests in addition to obtaining information about the actual previous level of functioning is a crucial part of the neuropsychological examination (Lezak et al., 2004). When assessing MCI, it is therefore considered necessary for the neuropsychologist to ensure that, in addition to using tests in recommended cognitive domains, tests are also used that can provide an indication of the original/premorbid cognitive function level.

## Norms

The current diagnostic systems for diagnosing MCI (NIA-AA, DSM-5, and ICD-11) require the use of age-, education-corrected, national and culturally adapted norms. Several normative studies have documented that this is necessary for valid assessment of cognitive function (e.g., Heaton et al., 2004; Cherner et al., 2020; Kiselica et al., 2024). However, many of the tests used in different countries may not meet such requirements. To address this problem in Norway and Sweden regarding diagnosing MCI, a multicenter study (Dementia Disease Initiation: Fladby et al., 2017) which investigates predictors for the development of neurodegenerative diseases, recently published Norwegian and Scandinavian norms based on neurologically healthy individuals. The norms are corrected for demographic variables such as age, gender and education, and cover all 5 cognitive domains that are recommended for examination. When compared with original norms, differences have emerged that can contribute to misdiagnosis when using original norms. This applies to all tests that have compared Norwegian and Scandinavian norms with original (often American) norms. There is therefore reason to assume that this will also apply to other neuropsychological tests where only original foreign norms are available. The findings are discussed in detail in the publications referenced below. In most of the publications there are online links to scoring calculators for the tests, which may be of interest especially for Scandinavian readers:

Learning and memory: Rey Auditory Verbal and Learning Test (RAVLT) (Espenes et al., 2023) and Rey Complex Figure Test - memory (RCFT) (Öhman et al., 2023). Attention/psychomotor speed: Trail Making Test A (TMT-A) (Espenes et al., 2020). Stroop -DKEFS Color Naming and Reading (Espenes et al., 2023). Language: Phonemic word fluency (FAS) (Lorentzen et al., 2023). Visuospatial function: Rey Complex Figure Copying Test (RCFT) (Öhman et al., 2023) and VOSP silhouettes (Eliassen et al., 2020). Complex attention/Executive function: Trail Making Test B (TMT-B) (Espenes et al., 2020) and Stroop -DKEFS Selective attention/response inhibition and response inhibition/alternating (Espenes et al., 2023).

The common practice in the interpretation of neuropsychological test scores involves relying on demographic normative data and make inferences about potential underlying pathology, without delving into whether the normative ‘pathological’ ranges are validated and truly hold diagnostic significance. Ilardi et al. (2024) point out that the conventional psychometric approaches to extract normative cutoffs may render neuropsychological tools inadequately sensitive for MCI.

## Norms for change

In connection with the assessment and follow-up of people with MCI, it is often appropriate to conduct a follow-up examination to measure any change in cognitive function over time. The traditional way to do this is to re-examine the patient after a time interval with the same tests as during the initial examination. A well-known problem with this method is the learning effect of repeated neuropsychological testing, which can help to camouflage any decline in cognitive function, which is common in neurodegenerative diseases. To better address this problem, 2-year cognitive change norms (Eliassen et al., 2023) have recently been published for key neuropsychological tests based on healthy controls from the Dementia Disease Initiation study, the Trønderbrain study and the Gothenburg MCI study (Fladby et al., 2017; Wallin et al., 2016; Grøntvedt et al., 2020). For more precise measurement of change over time than conventional re-testing, these norms are recommended for patients who are followed up after around 2 years. An online change norm calculator is included in Eliassen et al. (2023).

## Summary and recommendations

For the foreseeable future, the diagnostic systems DSM-5 and ICD-11 will be decisive for clinical practice and research in diagnosing MCI.

### Norms

Both DSM-5 and ICD-11 require neuropsychological/cognitive examination with tests and norms that are demographically and culturally adapted to the patient being examined. Many of the tests traditionally used in many countries may not meet these requirements. Based on the referenced normative studies, it has been shown that use of the tests’ original foreign norms can contribute to incorrect assessment of patients’ cognitive function when they are used in other countries and regions than where they were developed. On this basis, it is recommended that local or national norms, to the greatest extent possible, be used in diagnosing MCI.

### Neuropsychological testing in the assessment of MCI

As described, there may be weaknesses in short neuropsychological examinations that are based on only one test in each cognitive domain about sensitivity and specificity regarding brain-related cognitive impairment. For this reason, among other things, many studies have shown that MCI can be an unstable condition in which a high proportion of those who receive this diagnosis have normal neuropsychological function upon re-examination (Loewenstein et al., 2009; Overton et al., 2019). The neuropsychological method, which is based on a broader examination with at least two tests in each cognitive domain (where the failure criterion essentially requires two tests below the threshold in a domain for function in that domain to be considered impaired) have proven superior to cognitive screening. This method has been described by Jak et al. (2009) and later validated against various biomarkers and relationship to the development of cognitive impairment and dementia in studies with different populations (Bondi et al., 2014; Edmonds et al., 2016; Wong et al., 2018; Edmonds et al., 2020; Eliassen et al., 2017). While the comprehensive method have proven superior to cognitive screening, cognitive screeners should not be fully dismissed, as they are less time consuming, require less expertise (can usually be conducted by non-neuropsychologists), are less costly and have diagnostic potential despite their shortcomings (Fladby et al., 2017). However, there is reason to recommend that neuropsychologists who assess MCI use a neuropsychological method (for example Jak et al., 2009) using more than one test in each of the five cognitive domains being examined. Neither DSM-5 nor ICD-11 specify what kind of tests should be used or how extensive the examination should be. Studies that have validated the neuropsychological method for MCI diagnosis (Jak et al., 2009) have listed tests that have proven useful in diagnosing MCI (Table 1). It is also recommended to use tests that are usually less vulnerable to changes in brain function as indicators of initial/premorbid cognitive function level (Lezak et al., 2004). Among the tests in this category is the word comprehension subtest from the WAIS (Wechsler, 2008) as well as the National Adult Reading Test (NART), a widely accepted and commonly used method in clinical settings for estimating premorbid intelligence levels (Bright et al., 2018).

### Diagnostic algorithm

The Winblad criteria (Winblad et al., 2004) have proven to be clinically and research-wise useful over time. The criteria divide MCI into subcategories based on which cognitive domains are affected: amnestic single-domain and amnestic multiple-domain or non- amnestic single-domain and non -amnestic multiple-domain. This subcategorization of mild cognitive impairment is widespread throughout the world and helps to roughly divide cognitive impairment into clinically meaningful categories associated with different etiologies.

### Failure criterion

It is well documented that liberal cutoff values, around 1 standard deviation below the mean based on demographic norms, best balance sensitivity and specificity regarding underlying disease or pathology in the brain (Heaton et al., 2004; Taylor and Heaton, 2001; Jak et al., 2009; Bondi et al., 2014). At the same time, such test scores occur frequently in neurologically healthy individuals (Binder et al., 2009) and can be problematic to use as an indication of brain impairment. However, as part of a testing pattern, with more than one test in the same cognitive domain below 1 standard deviation below the mean, many studies have found this to be a good failure criterion for diagnosing MCI (Jak et al., 2009; Bondi et al., 2014). The leading diagnostic systems for MCI (NIA-AA, Albert et al., 2011; American Psychiatric Association, 2013) do not operate with any sharp dichotomization between normal and abnormal functioning but indicate typical test performance for MCI. For MCI, the NIA-AA (Albert et al., 2011) states that scores on cognitive tests are typically 1 to 1.5 SD below the mean of what is expected based on age-, education- and culturally appropriate norm data. The DSM-5 has chosen a similar approach, stating that test performance in MCI (mild neurocognitive disorder) is typically in the range of 1 to 2 standard deviations below the mean based on appropriate age-, education- and culturally appropriate norms. It is emphasized that these are considered typical ranges for MCI functioning and should not be considered strict cut-off values. Instead of absolute cutoffs, these diagnostic systems (NIA-AA, DSM-5, and ICD 11) advocate for a more individualized assessment that incorporates all available clinical data into the diagnostic process, including scores in a typical range for MCI, rather than scores below a specific limit or cutoff.

## What can the conclusion from the neuropsychological examination be used for when asking about MCI?

In the diagnosis of MCI, the neuropsychological examination represents the first objectification of cognitive dysfunction and thus has a central role in diagnostics and counseling for patients, relatives and healthcare professionals. Broadly speaking, the clinical neuropsychological examination can result in three different conclusions: 1. Positive neuropsychological findings, 2. Slight neuropsychological findings, of uncertain meaning, and 3. Normal neuropsychological function. Below are comments on the possible consequences of these conclusions.

### Positive neuropsychological findings

1. Such a conclusion assumes that the test results are considered valid and that they are within the clinical range according to current diagnostic systems.
2. The next step is to determine the cause of cognitive impairment. Detailed information about the development of cognitive impairment over time from the patient, preferably supported by information from an informant, is crucial as MCI presupposes a decline in cognitive function from a previous level of function. Psychological differential diagnoses such as anxiety, depression or more serious mental illness must be excluded as a likely cause of the neuropsychological findings. Furthermore, it may be appropriate to investigate the patient with biomarkers, including brain imaging, which may be associated with various forms of cognitive impairment and dementia. It is not uncommon for patients to be skeptical of biomarker testing as they fear that positive findings will indicate that they are in the process of developing dementia. This is understandable and must be respected, but it is also important to inform the patient that understanding the cause is a prerequisite for measures/intervention. Although there are currently no entirely safe treatments for Alzheimer’s disease, cognitive impairment of other causes can often be influenced by intervention. This may be relevant if the cognitive impairment is associated with mental illness, high blood pressure or another underlying medical condition that can be treated. Only after testing with various biomarkers has been done, is it possible to make a more detailed assessment of whether MCI is influenced by neurodegenerative conditions, vascular conditions or other medical conditions (for example heart failure, diabetes or cancer). As mentioned above Figure 1 (Winblad et al., 2004) amnestic MCI (both single and multiple domain) may raise suspicion of a degenerative cause, a possible early symptom of Alzheimer’s disease, while non- amnestic forms of MCI/mild neurodegenerative disorder to a greater extent raise suspicion of causes other than Alzheimer’s disease, including early phase of frontotemporal dementia, dementia with Lewy bodies or be an expression of depression or anxiety.
3. If the patient has MCI where a neurodegenerative cause is considered likely, the patient should be followed up with both medical and cognitive/neuropsychological re-examination. This can be done by a new neuropsychological examination, like the examination used in the first examination. With such a method, there is a risk that the results of the second examination will be influenced by learning effects and thus may give an incorrect picture of change in function and of current cognitive function. For more precise measurement of change over time than conventional re-testing, it is therefore recommended to use change norms (Eliassen et al., 2020) to the extent that such norms exist for the current re-test interval and tests with which the patient has been examined.

### Slight neuropsychological findings, of uncertain meaning

1. Slight neuropsychological findings, of uncertain meaning, are a characteristic that can be used if the examination only shows mildly reduced sporadic test results, without a test pattern that provides convincing support for the existence of a definite reduction in cognitive functions. This is a characteristic that is relevant for many patients who have undergone neuropsychological examination. This is natural because many studies have shown that “abnormal” performance on parts of a neuropsychological test battery occurs so frequently that it is psychometrically normal (Heaton et al., 2004; Binder et al., 2009). The characteristic implies ambiguity, something that one would prefer to avoid, but which may nevertheless be necessary to convey. In communication with the patient, depending on other anamnestic and medical information, it may either be correct to emphasize that the results represent normality, or to suggest uncertainty that justifies further investigation with biological markers and possible follow-up with re-examination if symptoms persist.

### Normal neuropsychological function

1. Patients who achieve normal function on neuropsychological testing generally have normal brain function (Taylor and Heaton, 2001). Individuals with high brain reserve and cognitive reserve (Stern, 2012) are more resistant to both brain and cognitive changes, and can have good cognitive performance even with underlying brain pathology. These represent a minority, and it is generally considered important to communicate to patients that normal neuropsychological test results are a good prognostic finding (Hessen et al., 2017; Eckerström et al., 2017)
2. Subjective cognitive impairment or subjective cognitive decline (SCD) (Jessen et al., 2014) is a common characteristic of patients who have experienced a decline or reduction in cognitive function in everyday life over time, but who perform normally on neuropsychological testing. People with SCD are of great research interest because it is thought that neurodegeneration can begin many years before it is possible to measure/objectify cognitive decline and brain changes, and that early interventions aimed at slowing the development of dementia should start at this level before irreversible changes in the brain and cognitive function have occurred. The prevalence of SCD in the older population is estimated to be around 25%, but varies widely from study to study, from 6–7 to 52%, partly because the different studies report from different cultures, demographic conditions and because they use different measurement instruments to map SCD (Röhr et al., 2020). Population studies suggest that people who experience subjective cognitive impairment have a slightly increased risk of developing dementia from a longitudinal perspective. For example, a large Swedish population study found that people over 60 years of age who reported memory difficulties had a higher risk of developing dementia in a 10-year perspective than people who did not report memory difficulties (Rönnlund et al., 2015). Another large study looked at the incidence of dementia development in people with SCD versus people without SCD and found that the incidence of Alzheimer’s dementia in people with SCD was 17.7/1000 person-years compared to 14.2 in control people without SCD. The corresponding figure for non-Alzheimer’s dementia was 6.1 versus 4.1. The risk of dementia increased significantly if people with SCD were recruited from a memory clinic, and even more so if the person had a low score on the MMSE and the presence of APOE-4 (Slot et al., 2019). This finding suggests that SCD in the population is often a relatively benign condition, while SCD in a clinical context is more associated with disease progression, consistent with 4–6 years of follow-up of memory clinic patients. In parallel studies with partly overlapping patient material, Hessen et al. (2017) found that SCD at 6 years of follow-up was a mainly benign condition with normal neuropsychology and absence of pathological biomarkers in spinal fluid at baseline, while Eckerström et al. (2017) found at 4 years of follow-up that patients with normal neuropsychology and pathological values of biomarkers in spinal fluid at baseline had a higher risk of developing cognitive impairment and dementia.

In conclusion, MCI is a condition that involves cognitive impairment beyond that expected with normal aging. It is a frequent condition, occurring in 23.7% of geriatric population world-wide (Salari et al., 2025). This condition may be a risk factor for the development of dementia. However, MCI can have medical and psychological causes that do not cause further cognitive decline or dementia. Thus, it is important to identify MCI at an early stage, aiming to prevent further impairment, to inform necessary life adaptation to cognitive problems or to treat the condition when the cause of cognitive impairment can be treated. The prognosis of MCI is often uncertain, partly caused by variable etiologies and partly caused by commonly employed brief and incomplete cognitive assessment. More comprehensive neuropsychological assessment based on age-, education-corrected, national and culturally adapted norms has shown superior validity regarding neuropathology and prognosis and is recommended as best practice. Furthermore, recent studies (Ilardi et al., 2024) have questioned the neuropathological validity regarding MCI and Alzheimer’s disease of commonly employed normative ‘pathological’ ranges, a question that needs further investigation for improvement of clinical practice.

## Correction note

**19 November 2025** This article has been corrected with minor changes. These changes do not impact the scientific content of the article.

**25 November 2025** This article has been corrected with minor changes. These changes do not impact the scientific content of the article.

**13 May 2026** This article has been corrected with minor changes. These changes do not impact the scientific content of the article.

**15 May 2026** This article has been corrected with minor changes. These changes do not impact the scientific content of the article.

**08 June 2026** This article has been corrected with minor changes. These changes do not impact the scientific content of the article.
